# Small-Bowel Perforation Caused by Multiple Metastases from Lung Adenocarcinoma Preceded by Obscure Gastrointestinal Bleeding: A Case Report

**DOI:** 10.70352/scrj.cr.26-0554

**Published:** 2026-09-16

**Authors:** Yusuke Hagiwara, Junichi Ochi, Saki Ohishi, Hiroshi Yokoyama, Norihito Kubo, Tsuyoshi Nozaki, Kazuhito Matsumoto, Naoki Wajima

**Affiliations:** 1 Department of Surgery, Odate Municipal General Hospital, Odate, Akita, Japan; 2 Department of Respiratory Medicine, Odate Municipal General Hospital, Odate, Akita, Japan; 3 Department of Pathology, Odate Municipal General Hospital, Odate, Akita, Japan

**Keywords:** lung adenocarcinoma, small-bowel metastasis, gastrointestinal perforation, obscure gastrointestinal bleeding, immune checkpoint inhibitor

## Abstract

**INTRODUCTION:**

Gastrointestinal metastasis from lung cancer is uncommon and rarely symptomatic, and small-bowel metastasis causing perforation is rare with a poor prognosis. Obscure gastrointestinal bleeding as the initial manifestation, preceding perforation, is seldom documented. We report such a case requiring emergency surgery.

**CASE PRESENTATION:**

A man in his 70s with a heavy smoking history was incidentally found, during an admission for bowel obstruction, to have left-lung lesions with hilar and subcarinal lymphadenopathy on CT, without abdominal disease. Bronchoscopic biopsy showed adenocarcinoma with a programmed death ligand 1 (PD-L1) tumor proportion score of 100% and no actionable driver alteration. Based on an ipsilateral separate-lobe pulmonary nodule, mediastinal nodal involvement, and a clinically suspected sacral bone metastasis, the disease was staged as cT4N2M1b, stage IVA. First-line pembrolizumab monotherapy was administered, and the thoracic disease remained controlled, with regression of the primary lesion and lymph nodes. After a cutaneous immune-related adverse event treated with corticosteroids (with pembrolizumab discontinuation), he developed melena and severe iron-deficiency anemia requiring transfusion. Esophagogastroduodenoscopy and colonoscopy identified no bleeding source, and obscure (suspected small-bowel) gastrointestinal bleeding was diagnosed. Approximately 2 months later, after an episode of shock, possibly related to infection and adrenal insufficiency following corticosteroid withdrawal, he developed acute abdominal pain with free intraperitoneal air. Emergency surgery revealed multiple small-bowel tumors with a perforation; partial small-bowel resection with functional end-to-end anastomosis and peritoneal lavage was performed. Histopathology showed multiple metastatic adenocarcinomas of the small intestine, a perforated metastatic ulcer, and mesenteric lymph node metastasis. Immunohistochemistry showed cytokeratin (CK) 7 positivity and CK20, caudal-type homeobox 2 (CDX2), thyroid transcription factor-1 (TTF-1), novel aspartic proteinase of the pepsin family A (Napsin A), CK5/6, and p40 negativity, matching the immunophenotype of the original lung biopsy. These findings were inconsistent with an intestinal primary and consistent with metastatic lung adenocarcinoma. Postoperative paralytic ileus resolved, and the patient was discharged on best supportive care.

**CONCLUSIONS:**

In patients with lung cancer, obscure gastrointestinal bleeding may herald small-bowel metastasis that can progress abruptly to perforation, even when the thoracic disease remains controlled after immune checkpoint inhibitor (ICI) therapy. Small-bowel metastasis should be considered when endoscopy is unrevealing, and new gastrointestinal symptoms should not be dismissed.

## Abbreviations

CDX2
: caudal-type homeobox 2
CK
: cytokeratin
CYFRA 21-1
: cytokeratin 19 fragment
FDG
: 18F-fluorodeoxyglucose
ICI
: immune checkpoint inhibitor
Napsin A
: novel aspartic proteinase of the pepsin family A
PD-L1
: programmed death ligand 1
SUVmax
: maximum standardized uptake value
TTF-1
: thyroid transcription factor-1

## INTRODUCTION

Gastrointestinal metastasis from lung cancer is detected in 2%–14% of autopsy series but rarely becomes clinically apparent.^1,2)^ When symptomatic, the small bowel is among the most frequently involved sites and may present with bleeding, obstruction, intussusception, or perforation.^3,4)^ Perforation is one of the most serious presentations; only a limited number of cases have been reported in the English literature, and the prognosis is dismal, with reported survival typically measured in weeks to a few months.^3,5)^ Because such lesions lie in the mid-gastrointestinal tract, beyond the reach of conventional upper and lower endoscopy, they are easily overlooked until a complication develops.

We report a case in which obscure gastrointestinal bleeding preceded metastatic small-bowel perforation requiring emergency surgery.

## CASE PRESENTATION

A man in his 70s with a 50-pack-year smoking history was admitted for bowel obstruction. His medical history included paroxysmal atrial fibrillation, a prior myocardial infarction, and previous surgery for intestinal volvulus. CT performed during the admission incidentally revealed lesions in the left lung—a cavitary, pleura-based lesion in S3 (29.4 mm) and a spiculated nodule in S8 (12 mm)—together with left hilar and subcarinal lymphadenopathy; no abdominal mass or visceral lesion was identified at that time. Bronchoscopic biopsy of the left upper lobe yielded adenocarcinoma. The tumor showed a PD-L1 tumor proportion score of 100% (22C3 assay), and comprehensive genomic profiling demonstrated no actionable driver alteration (a *KRAS* G12V variant was detected). Brain MRI showed no metastasis. FDG-PET/CT demonstrated intense uptake in the left S3 lesion (SUVmax 24.4), the left hilar and subcarinal lymph nodes, and the left S8 nodule (SUVmax 2.8); mild uptake was also observed in a sacral lesion (SUVmax 3.6), which was clinically suspected to represent bone metastasis. No abnormal uptake suggestive of small-bowel or other abdominal metastasis was reported. At diagnosis, serum carcinoembryonic antigen was within the normal range (4.3 ng/mL), CYFRA 21-1 was elevated (6.7 ng/mL), and the white blood cell count was 8.1 × 10^3^/μL. The patient was clinically staged as cT4N2M1b, stage IVA lung adenocarcinoma. As the disease was not amenable to curative-intent local therapy, it was managed as PD-L1–high, metastatic (stage IVA) lung adenocarcinoma with palliative systemic therapy.

**Fig. 1:**
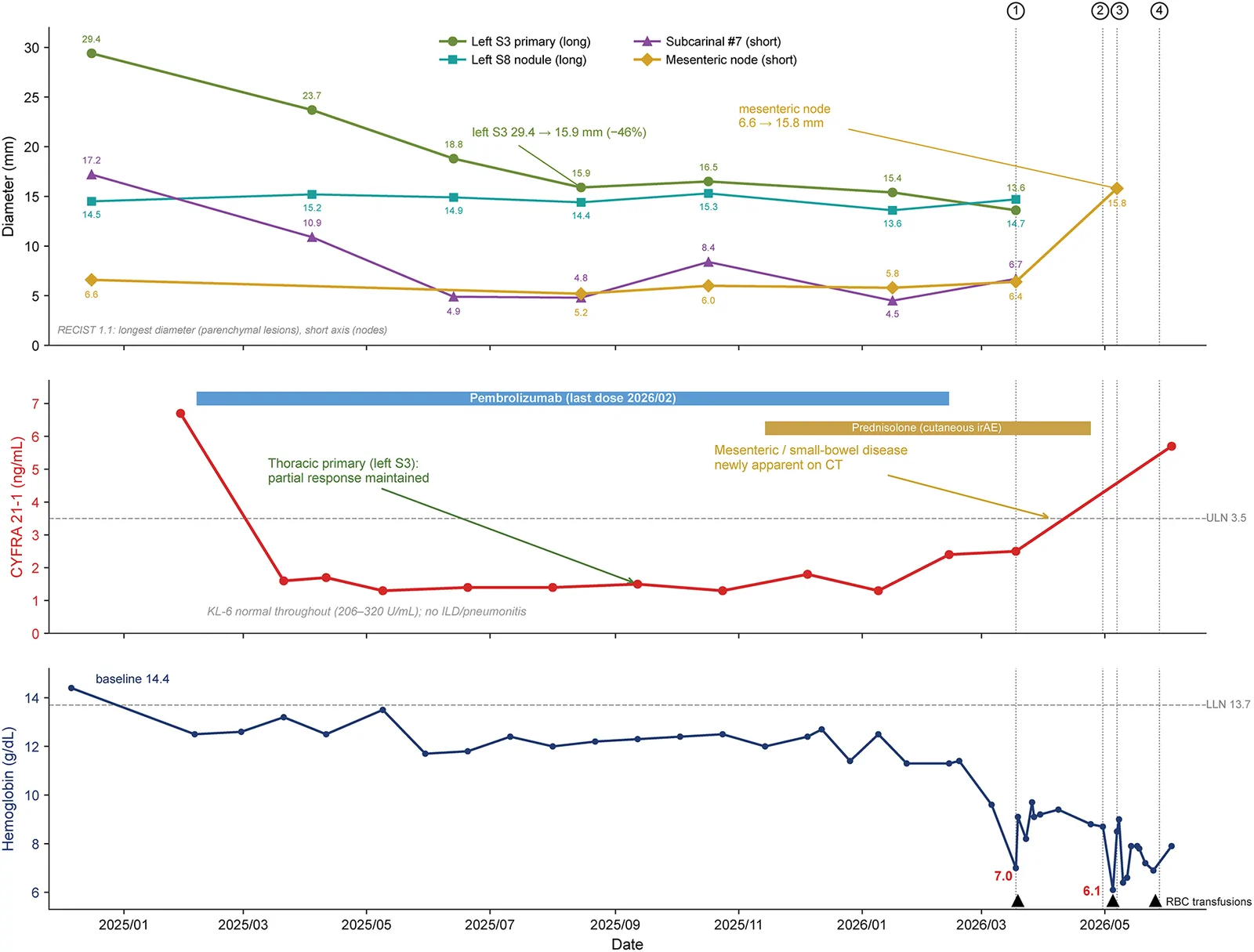
Integrated Clinical Timeline. Target-lesion diameters (top), serum CYFRA 21-1 (middle), and hemoglobin (bottom) are plotted on a shared time axis. Tumor diameters were measured according to RECIST 1.1 (longest diameter for parenchymal lesions and short axis for lymph nodes). Horizontal bars indicate the pembrolizumab and prednisolone treatment periods; dashed lines denote the upper limit of normal for CYFRA 21-1 and the lower limit of normal for hemoglobin, and inverted triangles indicate red-cell transfusions. Under pembrolizumab, the thoracic primary regressed (left S3, 29.4 mm→15.9 mm) and CYFRA 21-1 normalized, whereas mesenteric and small-bowel disease became apparent later (mesenteric lymph node, 6.6 mm→15.8 mm) with a concurrent rise in CYFRA 21-1. Hemoglobin declined to 7.0 g/dL in association with obscure gastrointestinal bleeding and again to 6.1 g/dL perioperatively, culminating in small-bowel perforation. Numbered markers denote: (1) gastrointestinal bleeding; (2) shock with suspected adrenal insufficiency; (3) small-bowel perforation with emergency resection; and (4) discharge on best supportive care. CYFRA 21-1, cytokeratin 19 fragment; irAE, immune-related adverse event; KL-6, sialylated carbohydrate antigen KL-6; RECIST, response evaluation criteria in solid tumors; LLN, lower limit of normal; ULN, upper limit of normal

**Fig. 2:**
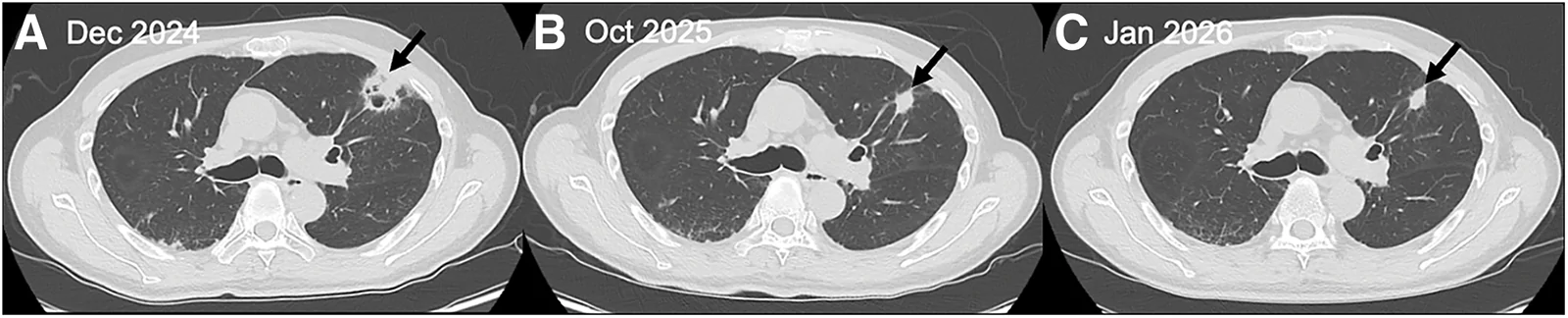
Serial Chest CT. Chest CT images obtained at baseline (**A**; December 2024), during pembrolizumab therapy (**B**; October 2025), and before the onset of gastrointestinal bleeding (**C**; January 2026). The arrows indicate the left S3 lung lesion, which showed reduction followed by sustained control.

First-line pembrolizumab monotherapy was started. On serial imaging, the left S3 primary regressed (longest diameter from 29.4 mm to 15.9 mm), the hilar and subcarinal nodes nearly resolved, and the thoracic disease remained controlled thereafter (**Figs. 1** and **2**); serial abdominal imaging obtained during this period showed no intra-abdominal disease. Serum CYFRA 21-1 normalized in parallel with the thoracic response (**Fig. 1**).

Approximately 9 months into treatment, the patient developed a cutaneous eruption diagnosed as a grade 2 cutaneous immune-related adverse event. Pembrolizumab was withheld and oral prednisolone was started and gradually tapered, with recurrent flares requiring repeated dose adjustments; pembrolizumab was ultimately discontinued after approximately 1 year of therapy because of the persistent skin reaction.

About 1 month after the last dose of pembrolizumab (approximately 13 months after starting therapy), the patient developed melena and dizziness, with severe iron-deficiency anemia (hemoglobin 7.0 g/dL; iron-deficiency pattern: serum iron 12 μg/dL, ferritin 8.8 ng/mL, transferrin saturation 3%) requiring red-cell transfusion (**Fig. 1**). Esophagogastroduodenoscopy showed only candidiasis, a hiatal hernia, and gastritis, and total colonoscopy revealed no bleeding source. Obscure (suspected small-bowel) gastrointestinal bleeding was diagnosed; antiplatelet and anticoagulant agents were discontinued and oral iron was started.

Approximately 1 week after the corticosteroid was discontinued, and following several days of appetite loss, the patient collapsed and was admitted in a state of shock. He was treated empirically for possible infection with vasopressors and broad-spectrum antibiotics, and suspected adrenal insufficiency related to corticosteroid withdrawal was treated with stress-dose hydrocortisone. Anemia recurred and required further transfusion.

**Fig. 3:**
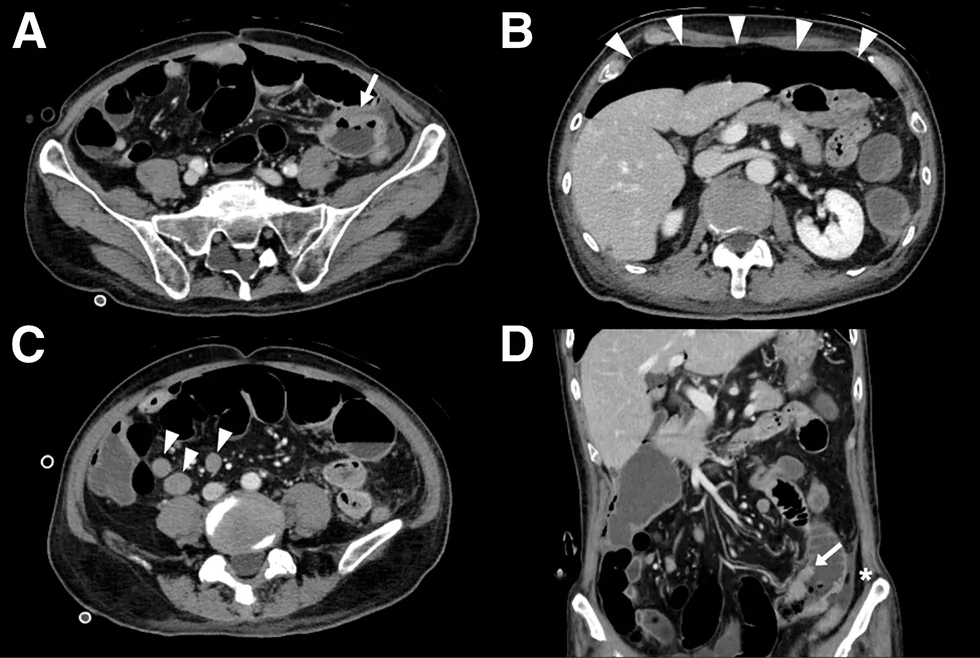
CT at Small-Bowel Perforation. (**A**) Axial image showing localized, contrast-enhancing wall thickening of the small bowel (arrow). (**B**) Axial image showing intraperitoneal free air (arrowheads). (**C**) Axial image showing enlarged mesenteric lymph nodes (arrowheads). (**D**) Coronal image showing the relationship between the small-bowel lesion (arrow) and ascites (asterisk).

**Fig. 4:**
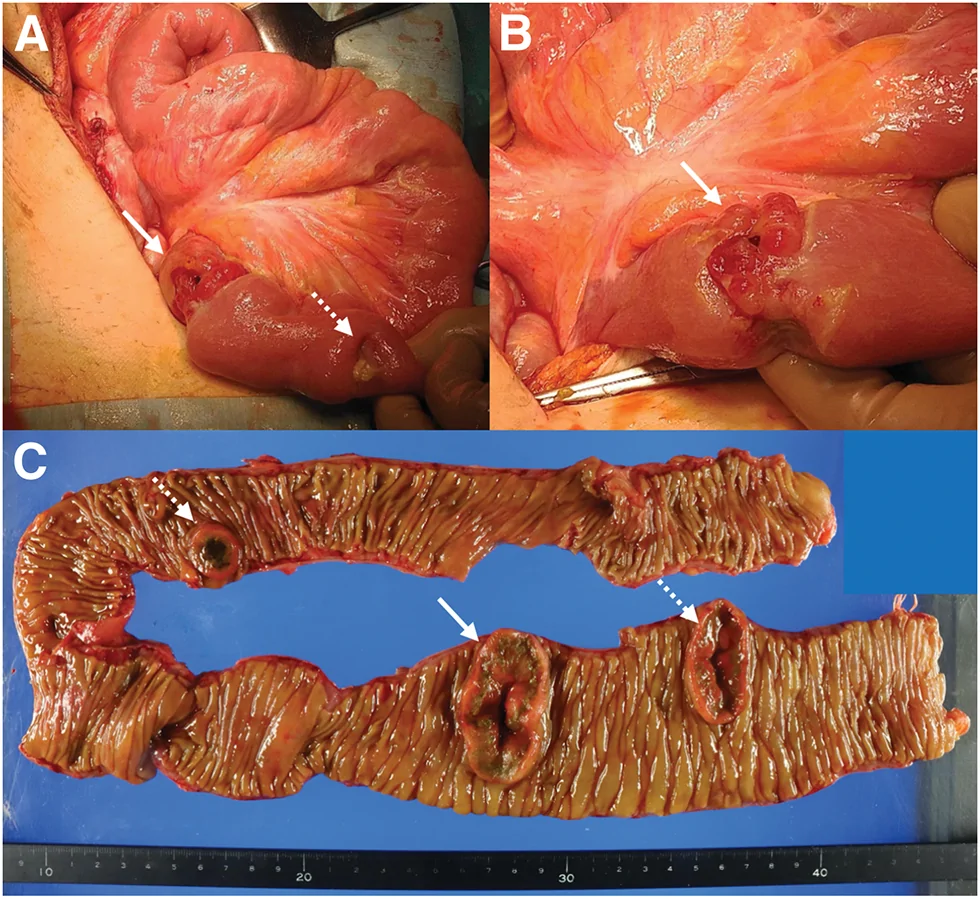
Intraoperative and Resected-Specimen Findings. (**A**) Intraoperative photograph showing the perforation site (solid arrow; 100 cm distal to the ligament of Treitz) and a non-perforated lesion with serosal irregularity and puckering (dashed arrow; 90 cm distal to the ligament of Treitz). (**B**) Magnified intraoperative view of the perforation site. (**C**) Macroscopic view of the freshly resected specimen, opened to expose the luminal surface, showing the perforated tumor lesion (solid arrow) and additional non-perforated tumor lesions (dashed arrows).

A few days later—approximately 2 months after the onset of the obscure gastrointestinal bleeding—he developed acute abdominal pain, and CT demonstrated free intraperitoneal air together with small-bowel wall thickening and an enlarging mesenteric lymph node (short axis 6.6 mm–15.8 mm), indicating gastrointestinal perforation (**Fig. 3**). At emergency laparotomy, multiple small-bowel tumors were identified at several sites distal to the ligament of Treitz; the perforated lesion lay approximately 100 cm, and a non-perforated lesion with serosal puckering approximately 90 cm, distal to the ligament of Treitz. The perforated lesion showed a serosa-exposed tumor with a perforation approximately 4 mm in diameter and enteric leakage, and mesenteric nodules were present (**Fig. 4**). A partial small-bowel resection including the perforation site was performed with functional end-to-end anastomosis, followed by peritoneal lavage and drainage; the procedure was palliative.

**Fig. 5:**
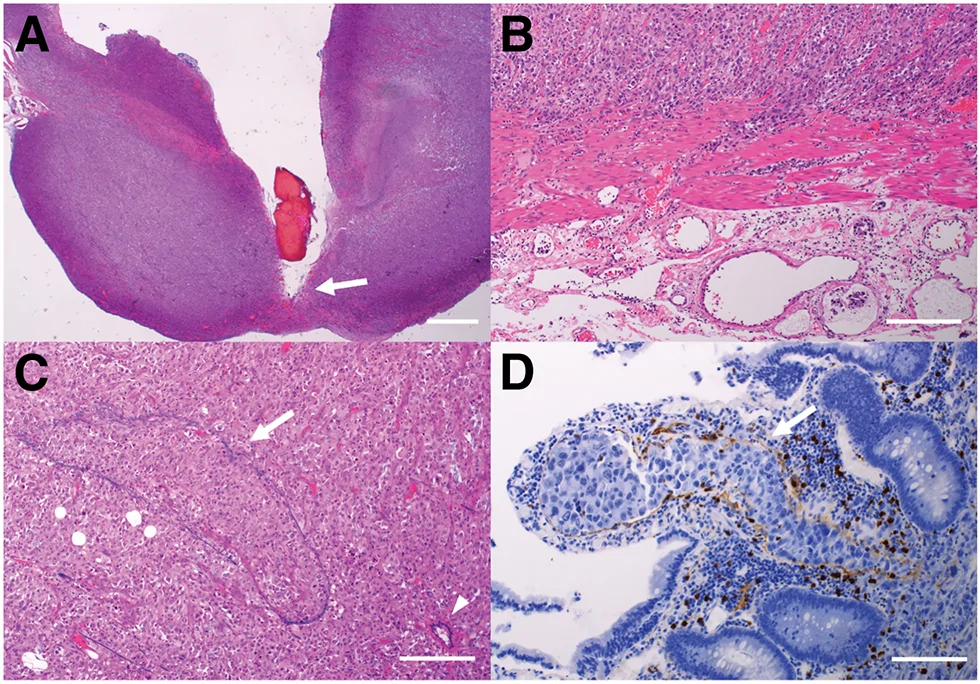
Histopathological Findings of the Small-Bowel Metastasis. (**A**) Low-power hematoxylin and eosin staining showing central tumor perforation with transmural involvement of the bowel wall. (**B**) Hematoxylin and eosin staining showing irregular solid proliferation of poorly differentiated adenocarcinoma invading beyond the muscularis propria into the subserosal layer. (**C**) Victoria blue–hematoxylin and eosin (elastica) staining showing venous invasion, with tumor cells filling the lumen of an elastic-walled vein (arrow); the adjacent artery (arrowhead) is uninvolved. (**D**) D2-40 immunostaining showing lymphatic invasion, with tumor cells filling a D2-40-positive lymphatic channel (arrow) beneath preserved small-bowel epithelium. Scale bars: 1000 μm (**A**), 200 μm (**B**, **C**), and 100 μm (**D**).

**Fig. 6:**
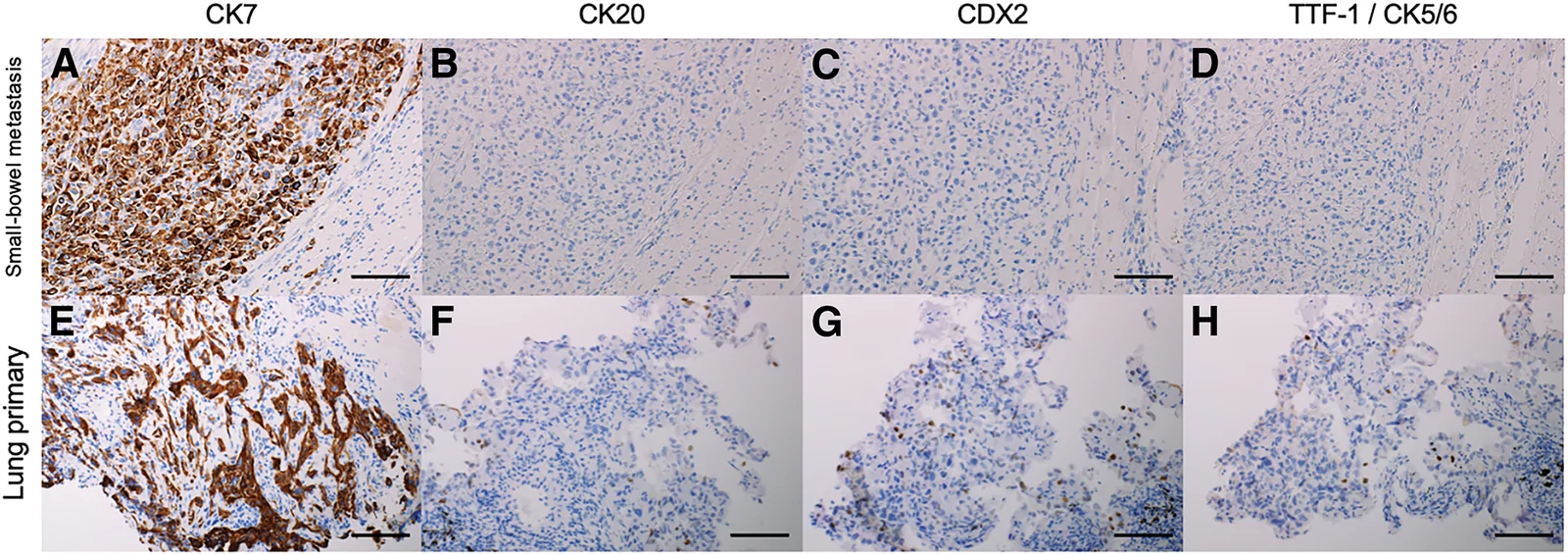
Comparative Immunohistochemistry of the Small-Bowel Metastasis and the Original Lung Biopsy. Upper row (**A**–**D**), small-bowel metastasis; lower row (**E**–**H**), original lung biopsy. (**A**, **E**) CK7 positivity. (**B**, **F**) CK20 negativity. (**C**, **G**) CDX2 negativity. (**D**, **H**) Negativity for a TTF-1 (nuclear)/CK5/6 (cytoplasmic) cocktail. Napsin A and p40, assessed as a separate p40 (nuclear)/Napsin A (cytoplasmic) cocktail, were also negative in the tumor cells of both lesions (**Supplementary Fig. S1**). The tumor cells of both the small-bowel metastasis and the lung primary shared an identical immunophenotype (CK7-positive; CK20-, CDX2-, TTF-1-, Napsin A-, CK5/6-, and p40-negative), supporting a diagnosis consistent with metastatic lung adenocarcinoma. Scale bars, 100 μm. CDX2, caudal-type homeobox 2; CK, cytokeratin; TTF-1, thyroid transcription factor-1

Histopathological examination showed multiple poorly differentiated adenocarcinomas involving the small intestine, with a perforated metastatic ulcer and metastasis in 1 of 3 regional mesenteric lymph nodes (**Fig. 5**). The tumors proliferated in an irregular solid pattern with transmural growth through the bowel wall, and the perforation was located at the center of 1 tumor. Venous invasion, highlighted by Victoria blue–hematoxylin and eosin (elastica) staining, and lymphatic invasion, highlighted by D2-40 immunostaining, were both identified (**Fig. 5**). Immunohistochemistry of the small-bowel tumor was positive for CK7 and negative for CK20, CDX2, TTF-1, Napsin A, CK5/6, and p40. Retrospective immunohistochemical staining of the original lung biopsy demonstrated an identical immunophenotype, with CK7 positivity and CK20, CDX2, TTF-1, Napsin A, CK5/6, and p40 negativity (**Fig. 6** and **Supplementary Fig. S1**). The transmural, multifocal growth with lymphovascular invasion and the pattern of mucosal involvement favored metastatic disease over an intestinal primary, and the immunophenotype concordant with the patient’s known pulmonary adenocarcinoma supported a diagnosis consistent with metastatic lung adenocarcinoma rather than a primary small-bowel cancer.

The early postoperative course was complicated by paralytic ileus, which resolved with conservative management, and oral intake was resumed. Given the multiplicity of the small-bowel metastases, the risk of rebleeding, and the overall condition, best supportive care was adopted after discussion with the patient and family. He was discharged in ambulatory condition approximately 3 weeks after surgery and remained alive at the latest follow-up, 27 days postoperatively. The patient noted that his gastrointestinal symptoms had been difficult to recognize before the emergency, and he consented to the publication of his case in the hope that it would benefit others.

## DISCUSSION

Gastrointestinal metastasis from lung cancer is uncommon in clinical practice, although it is reported in 2%–14% of autopsy series.^1,2)^ The small bowel is among the most frequently involved gastrointestinal sites,^4)^ yet symptomatic disease is rare, and perforation is one of its most serious manifestations. Only a limited number of cases of small-bowel perforation from metastatic lung cancer have been reported in the English literature, with the jejunum most commonly affected, and all major histological subtypes—including adenocarcinoma—have been implicated.^3,5)^ The prognosis is dismal, with reported survival typically on the order of weeks to a few months, and the course of the present patient, who was discharged on best supportive care, is consistent with this poor outlook.

The most instructive feature of this case is that the small-bowel metastases first became clinically apparent as obscure gastrointestinal bleeding—melena and iron-deficiency anemia despite unremarkable esophagogastroduodenoscopy and colonoscopy—and only later progressed to perforation, approximately 2 months later. Small-bowel metastases may ulcerate and bleed before reaching transmural necrosis, but lesions in the mid-gastrointestinal tract are beyond the reach of conventional endoscopy and are readily missed. In a patient with known lung cancer and obscure gastrointestinal bleeding with negative bidirectional endoscopy, small-bowel metastasis should be included in the differential diagnosis, and dedicated small-bowel evaluation—capsule endoscopy, device-assisted enteroscopy, or CT/MR enterography—should be considered when the clinical condition permits.^6,7)^ Recognizing this bleeding-before-perforation sequence may allow earlier diagnosis before a catastrophic abdominal event.

A further noteworthy aspect is the clinical context. The thoracic disease remained controlled during and after first-line pembrolizumab monotherapy for PD-L1–high advanced lung adenocarcinoma, whereas the small-bowel metastases became clinically evident and progressed to perforation. Heterogeneous, organ-specific responses to ICIs are increasingly recognized in non–small-cell lung cancer.^8)^

The first published case of small-bowel perforation from lung adenocarcinoma following pembrolizumab treatment was reported by Sato et al.,^9)^ in which perforation of a small-bowel metastasis from PD-L1–high lung adenocarcinoma occurred approximately 1 month after pembrolizumab was stopped for drug-induced pneumonitis. Our patient followed a similar post-discontinuation course: the obscure gastrointestinal bleeding and the subsequent perforation developed after pembrolizumab had been discontinued for a cutaneous immune-related adverse event, rather than during active ICI therapy. In contrast to that report, in which multiple metastases including small-bowel lesions were already known, the distinctive feature of the present case is that obscure gastrointestinal bleeding preceded overt perforation by approximately 2 months; although no abdominal lesion was evident on pretreatment FDG-PET/CT or CT, occult small-bowel metastasis at diagnosis cannot be completely excluded. The perforation most likely resulted from transmural tumor growth with central necrosis and ulceration, as seen histologically, rather than from an immune-mediated process. Gastrointestinal events during or after ICI therapy are not always immune-related adverse events. Kim et al.^10)^ attributed intestinal perforation to pseudoprogression during pembrolizumab, in contrast to the post-discontinuation course of our patient. Separately, a diffuse jejunal metastasis was initially misdiagnosed as ICI-associated enteritis.^11)^ Together with our case, these reports indicate that new gastrointestinal symptoms—including obscure bleeding—arising during or after ICI therapy should prompt consideration of small-bowel metastasis in addition to immune-related enteritis.

The origin of the small-bowel tumors was established by clinicopathological correlation. Morphologically, the tumors showed transmural, multifocal growth with frequent lymphovascular invasion and mesenteric nodal metastasis, a pattern more in keeping with metastatic spread than with a mucosa-based primary adenocarcinoma. CK7 positivity with CK20 and CDX2 negativity argues against a primary intestinal adenocarcinoma, in which CK20 and CDX2 are typically expressed, and is compatible with a non-gastrointestinal, including pulmonary, origin.^12,13)^ Although TTF-1 and Napsin A were negative in both the small-bowel metastasis and the lung primary, these markers have imperfect sensitivity for pulmonary adenocarcinoma, and their absence does not exclude a lung primary,^14,15)^ particularly in the presence of a biopsy-proven pulmonary adenocarcinoma, multiple synchronous small-bowel lesions with mesenteric nodal involvement, and no alternative primary. Most importantly, retrospective staining of the original lung biopsy demonstrated an identical immunophenotype, including CK7 positivity with CK20, CDX2, TTF-1, and Napsin A negativity; the negativity of these lineage markers therefore reflected the immunophenotype of this patient’s own pulmonary adenocarcinoma rather than arguing against a lung origin. Direct comparison with the primary tumor was thus more informative than reliance on any single marker, and taken together the findings were considered consistent with metastatic lung adenocarcinoma.

This report has several limitations. The diagnosis rests on morphology, immunohistochemistry, and clinical context rather than molecular confirmation; the driver profile was not matched between the primary and the metastasis, and the *KRAS* G12V alteration detected in the primary is not lineage-specific, occurring in both pulmonary and colorectal adenocarcinoma, so it would not by itself have been discriminatory. The small-bowel bleeding source was not localized preoperatively by capsule endoscopy, device-assisted enteroscopy, or angiography, and concurrent antithrombotic therapy may have contributed to the bleeding. Although no small-bowel metastasis was detectable on staging FDG-PET/CT and CT at diagnosis, occult micrometastasis cannot be excluded and the precise timing of metastatic seeding cannot be determined; moreover, the sacral lesion was clinically suspected on imaging but not pathologically confirmed. Finally, the perioperative course was complicated by an immune-related cutaneous adverse event and by suspected adrenal insufficiency following corticosteroid withdrawal, illustrating the management complexity that can accompany checkpoint-inhibitor therapy. Despite these limitations, the overall constellation of findings supports metastatic lung adenocarcinoma as the most likely diagnosis.

## CONCLUSIONS

In patients with lung cancer, obscure gastrointestinal bleeding may herald small-bowel metastasis, which can progress abruptly to perforation requiring emergency surgery. Small-bowel metastasis should be considered when upper and lower endoscopy are unrevealing, and dedicated small-bowel evaluation should be pursued when feasible. Importantly, new gastrointestinal symptoms should not be dismissed even when the thoracic disease remains controlled after ICI therapy, as clinically significant extrathoracic progression may still occur.

## SUPPLEMENTARY MATERIALS

Supplementary Fig. S1 p40/Napsin A Immunohistochemistry of the Small-Bowel Metastasis and the Original Lung Biopsy. A p40 (nuclear)/Napsin A (cytoplasmic) cocktail was negative in the tumor cells of both the small-bowel metastasis (**A**) and the original lung biopsy (**B**). Scattered Napsin A–positive non-neoplastic alveolar cells in (**B**) served as an internal positive control. Scale bars, 100 μm.

## DECLARATIONS

### Use of generative AI and AI-assisted technologies

During the preparation of this manuscript, the authors used ChatGPT (OpenAI) and Claude (Anthropic) to assist with language editing, formatting, and figure and cover-letter drafting. The authors reviewed and verified all content and take full responsibility for the manuscript.

### Availability of data and materials

The datasets supporting the conclusions of this article are available from the corresponding author on reasonable request.
